# Molecular analyses of glioblastoma stem-like cells and glioblastoma tissue

**DOI:** 10.1371/journal.pone.0234986

**Published:** 2020-07-07

**Authors:** Marco Wallenborn, Li-Xin Xu, Holger Kirsten, Leili Rohani, Daniela Rudolf, Peter Ahnert, Christian Schmidt, Ronny M. Schulz, Mandy Richter, Wolfgang Krupp, Wolf Mueller, Adiv A. Johnson, Jürgen Meixensberger, Heidrun Holland

**Affiliations:** 1 Translational Centre for Regenerative Medicine (TRM) and Saxonian Incubator for Clinical Translation (SIKT), University of Leipzig, Leipzig, Germany; 2 Department of Neurosurgery, University of Leipzig, Leipzig, Germany; 3 Institute for Medical Informatics, Statistics and Epidemiology (IMISE), University of Leipzig, Leipzig, Germany; 4 LIFE Research Centre for Civilization Diseases, University of Leipzig, Leipzig, Germany; 5 Department of Biochemistry and Molecular Biology, University of Calgary, Calgary, Canada; 6 Clinic of Orthopaedics, Traumatology and Plastic Surgery, Faculty of Medicine, University of Leipzig, Leipzig, Germany; 7 Department of Neuropathology, University of Leipzig, Leipzig, Germany; 8 Nikon Instruments, Melville, New York, United States of America; Sechenov First Medical University, RUSSIAN FEDERATION

## Abstract

Glioblastoma is a common, malignant brain tumor whose disease incidence increases with age. Glioblastoma stem-like cells (GSCs) are thought to contribute to cancer therapy resistance and to be responsible for tumor initiation, maintenance, and recurrence. This study utilizes both SNP array and gene expression profiling to better understand GSCs and their relation to malignant disease. Peripheral blood and primary glioblastoma tumor tissue were obtained from patients, the latter of which was used to generate GSCs as well as a CD133^pos.^/CD15^pos.^ subpopulation. The stem cell features of GSCs were confirmed via the immunofluorescent expression of Nestin, SOX2, and CD133. Both tumor tissue and the isolated primary cells shared unique abnormal genomic characteristics, including a gain of chromosome 7 as well as either a partial or complete loss of chromosome 10. Individual genomic differences were also observed, including the loss of chromosome 4 and segmental uniparental disomy of 9p24.3→p21.3 in GSCs. Gene expression profiling revealed 418 genes upregulated in tumor tissue vs. CD133^pos.^/CD15^pos.^ cells and 44 genes upregulated in CD133^pos.^/CD15^pos.^ cells vs. tumor tissue. Pathway analyses demonstrated that upregulated genes in CD133^pos.^/CD15^pos.^ cells are relevant to cell cycle processes and cancerogenesis. In summary, we detected previously undescribed genomic and gene expression differences when comparing tumor tissue and isolated stem-like subpopulations.

## Introduction

Glioblastoma is a common and highly aggressive, malignant primary brain tumor that is typically located in the cerebral hemispheres. Primary glioblastoma will often grow rapidly *de novo* absent of recognizable precursor lesions and is IDH-wildtype. In contrast, secondary glioblastoma is IDH-mutant and tends to develop slowly from diffuse astrocytoma [1]. Survival outcomes are poor with very few patients surviving to 2.5 years and less than 5% of patients surviving to 5 years following diagnosis [2]. Therefore, novel treatments and therapies are needed to improve the survival of patients diagnosed with glioblastoma.

Although there is no consensus on the existence of glioblastoma stem-like cells (GSCs) so far, many research endeavors are underway to identify features of glioblastoma cells with stem cell characteristics. Within tumors, cancer cells with stem cell characteristics (properties of multi-lineage differentiation and self-renewal) are thought to stimulate tumor growth, development, and recurrence. These cells have been hypothesized to drive long-term tumor growth as well as underlie poor outcomes of aggressive cancers like malignant glioma [3]. Unique to glioblastoma, GSCs seem to be functional subsets of cells that are radioresistant and chemoresistant. GSCs have been proposed as a clinical target to improve patient outcomes [4] and recent data suggest that the eradication of GSCs may be required to successfully treat glioblastoma patients [5].

Although little is known about this cell type, it is clear that they harbor unique molecular and cellular features. GSCs express markers like CD133, Nestin, and SOX2 [6–8] and, presently, no ideal set of markers exists to properly characterize GSCs. In the literature, different stem cell markers (e.g. SOX2) and cell surface markers (e.g. CD133) have been suggested to help enrich GSCs [9].

Work by Baronchelli et al. has shown that cytogenetic alterations are common in GSC cell lines. Specifically, copy number alterations in the *EGFR*, *RB1*, and *PTEN* genes were identified [10]. By analyzing public gene expression profile datasets, 336 switch genes were discovered that are potentially involved in the transition from an undifferentiated to a differentiated state in GSCs. Switch genes were those which showed up- or downregulation in gene expression. A subset of these genes were downregulated and relevant to the extracellular matrix as well as to focal adhesions [11]. Cell differentiation was also correlated with the downregulation of various transcription factors, including SOX2 [11]. Cell signaling is additionally perturbed in GSCs. For example, the Cox-2 and Wnt signaling pathways interact to maintain cancer stem cell identify and are aberrantly activated in this cell type [12]. Calcium signaling genes are also differentially expressed in quiescent and proliferating GSCs [13].

These findings demonstrate that GSCs exhibit unique genetic and molecular characteristics. The development of effective therapies targeting these cells will require the identification of robust, specific cellular markers, which will necessitate a much more comprehensive understanding of these cells. This study aims to promote these goals by comparing the molecular profiles of tumor tissue and tumor stem-like cell populations (GSCs and CD133^pos.^/CD15^pos.^ cells).

## Material and methods

### Study design

Five patients with glioblastoma surgery, performed by the Department of Neurosurgery, University Hospital of Leipzig, were included in the present study. Informed consent from the patients for surgery and this study were obtained. The study was approved by the Ethics Committee (086–2008) at the University of Leipzig. All patients were lacking a family history of brain tumors and the tumors were classified as primary glioblastoma WHO grade IV by the local Institute of Neuropathology at Leipzig University. The following immunohistochemical markers were analyzed: MIB-1 (Ki-67), GFAP, IDH-1 (R132H), Olig-2, and p53by the Department of Neuropathology, University Hospital of Leipzig.

Tumor tissue resection was performed for all patients. After surgery, neurological deficits were assessed for each patient. Table 1 summarizes detailed patient information.

**Table 1:** Individual characteristics of each patient. Detailed information on age, gender, tumor size and site, and the results of immunohistochemical examination are shown.

Sample ID	P1	P2	P3	P4	P5
**Age at Surgery (Years)**	63	84	56	72	59
**Gender**	Female	Female	Female	Male	Female
**Tumor Site**	Left parietal	Right frontal	Right parietal	Right frontal lobe	Right frontal
**Tumor Size (cm)**	5×2.5×2	2×1.5×0.5	1.2×1×0.2	5×4×1	2.5×1.5×0.3
**Overall Survival**	Unknown	7.5 months	Unknown	12 months	4 months
**MIB-1 (Ki-67)**	~30%	25–30%	~20%	≤6%	25–30%
**GFAP**	+++	++	+++	+++	++
**IDH-1 (R132H)**	-	-	-	-	-
**Olig-2**	+++	++	++	-	++
**p53**	~2% +++,	++	~15% +++,	≤25% +++	~5% ++
	~10% +/++		~20% ++		

GFAP–Glial fibrillary acidic protein; IDH1 –Isocitrate Dehydrogenase 1; “-”–Negative; “+”–Slightly Positive; “++”–Positive; “+++”–Strongly positive.

### Tumor tissue and peripheral blood preservation

Primary tumor tissue was dissociated mechanically and enzymatically using collagenase. Fresh tumor samples after surgical resection were preserved in DMEM/F-12 (Cat. 10565–018, Thermo Fisher Scientific, Waltham, Massachusetts, United States of America) and divided into pieces. Each piece was subsequently cryopreserved at -80°C in Recovery™ Cell Culture Freezing Medium (Cat. 12648–010, Thermo Fisher Scientific, Waltham, Massachusetts). These divided tissue samples were utilized to generate primary explant cell cultures, which were then transferred to primary serum-free cell culture. Peripheral blood was preserved in an EDTA tube (Cat. 05.1167, Sarstedt, Nümbrecht, Germany) or a Lithium-Heparin tube (Cat. 05.1553, Sarstedt, Nümbrecht, Germany). The blood samples were cryopreserved at -80°C (EDTA; whole blood was frozen) or cultured (Heparin) with Peripheral Blood Karyotyping Medium (Cat. 01-201-1B, Biological Industries, Beit Haemek, Israel).

### Explant cell culture (serum-contained cell culture)

Fresh non-necrotic tissue of glioblastoma mechanically disaggregated into small pieces. Cells were cultured with AmnioMAX™ C-100 Basal Medium (Cat. 17001–082, Thermo Fisher Scientific), supplemented with AmnioMAX™ C-100 Supplement (Cat. 12556–015, Thermo Fisher Scientific) and incubated at 37°C with 5% CO_2_. After tumor cell out-growth, cells were covered with supplemented AmnioMAX™ Basal Medium [14].

### Serum-free cell culture

The serum-free cell culture was carried out according to previously published methods [15–17]. Fresh, non-necrotic tumor tissue was minced and incubated at room temperature. The tissue fragments were washed, centrifuged, and resuspended in 1 mg/ml Collagenase-2 mM CaCl_2_-PBS working solution [Collagenase NB 4 (Cat. 17454.01, SERVA, Heidelberg, Germany), CaCl_2_ (Cat. C7902, Sigma-Aldrich, St. Louis, Missouri, United States of America) PBS] for an enzymatical dissociation at 37°C. Fragments were neutralized with 4 mM EDTA-PBS solution (Cat. 8040.2, Carl Roth, Karlsruhe, Germany). The cells were resuspended in a complete medium, consisted of DMEM/F-12 (Cat. 10565–018, Thermo Fisher Scientific) with B-27 (Cat. 12587–010, Thermo Fisher Scientific), N-2 (Cat. 17502–048, Thermo Fisher Scientific), GlutaMAX (Cat. 35050–038, Thermo Fisher Scientific), Penicillin-Streptomycin (100 U/ml, Cat. 15140–122, Thermo Fisher Scientific), EGF (20 ng/ml, Cat. AF-100-15, PeproTech, Rocky Hill, New Jersey), FGF-basic (20 ng/ml, Cat. AF-100-18B, PeproTech) and Heparin (5000 IE/ml, Cat. 03170642, Ratiopharm, Ulm, Germany). This suspension was incubated at 37°C with 5% CO_2_. The flasks were coated with 1 mg/cm^2^ of Laminin (Cat. L2020, Sigma-Aldrich) and incubated at 37°C with 5% CO_2_. The cell growth was monitored 2–3 times a week and passages were carried out by Accutase (Cat. A11105-01, Thermo Fisher Scientific) at 37°C.

### Immunofluorescence and immunohistochemistry of serum-free cultured cells

Cells of the serum-free culture were examined for stem cell markers by immunofluorescence prior to magnetic sorting. The previously seeded and cultured cells were fixed with 4% paraformaldehyde (Cat. 28906, Thermo Scientific) at 4°C. The cells were permeabilized with 0.5% Triton X-100 (Cat. K28857603, MERCK, Kenilworth, New Jersey, United States of America) in PBS and blocked with 10% Fetal Bovine Serum (Cat. 12483020, Thermo Fisher Scientific). Primary antibodies were mouse anti-human Nestin (5 μg/ml, Cat. 14–9843, Thermo Fisher Scientific), rabbit anti-human SOX2 (1:400, Cat. #3579, Cell Signaling Technology, Danvers, Massachusetts, United States of America) and mouse anti-human CD133 (1:10, Cat. 130-090-422, Miltenyi Biotec, Bergisch Gladbach, Germany). Secondary antibodies were donkey anti-rabbit and anti-mouse Alexa Fluor 488 (1:1000, Green, Cat. A21206 and A21202, Thermo Fisher Scientific) and donkey anti-mouse Alexa Fluor 546 (1:1000, Red, Cat. A10036, Thermo Fisher Scientific). After washing with PBS, the nucleus was stained with DAPI (1:10000, Cat. 06J50, Abbott, Chicago, Illinois, United States of America) and then further washed with PBS.

### Multi-parameter magnetic-activated cell sorting of serum-free cultured cells

The multi-parameter magnetic-activated cell sorting (MACS) was conducted to isolate tumor cells simultaneously carrying the stem cell markers CD15 and CD133. The procedures were performed according to manufacturer’s instructions with optimizations. Magnetic labeling of CD15 and CD133 was performed using the Anti-PE MultiSort Kit (Cat. 130-090-757, Miltenyi Biotec). After centrifugation, the cell pellet was resuspended and incubated with DNase I + MACS buffer [10 mg DNase I (Cat. 10104159001, Roche, Basel, Switzerland) dissolved in MACS buffer (Cat. 130-091-221, Miltenyi Biotec)], FcR Blocking Reagent (Cat. 130-059-901, Miltenyi Biotec), and CD15^neg.^ PE antibody (Cat. 130-098-010, Miltenyi Biotec) at 4°C. Cells were incubated with DNase I + MACS buffer and Anti-PE Multisort MicroBeads at 4°C. All fractions of cells led to the release of MicroBeads by applying MultiSort Release Reagent and DNase I + MACS buffer at 4°C. After magnetic separation, unsorted serum-free cultured cells (GSCs) and CD15^pos.^/CD133^pos.^ cells were centrifuged. The cells were cryopreserved in -80°C.

For evaluation of the cell separation by MACS technology, the cells were analyzed by flow cytometry using MACSQuant Analyzer 10 and MACSQuantify ™ 2.5 (Miltenyi Biotec GmbH) as well as FlowJo (10.07) Software for data analysis. The boundary between positive and negative cells was set based on isotype control mouse IgG1-PE clone IS5-21F5 (Miltenyi Biotec GmbH).

### DNA and RNA isolation

DNA isolation from blood samples was performed using QIAmp DNA Blood Mini Kit according to the protocol “DNA Purification from Blood or Body Fluids” (QIAGEN GmbH, Hilden, Germany). DNA and RNA were extracted from primary tumor tissue, tumor-derived serum free cell culture, and the MACS separated cell subpopulation using AllPrep DNA/RNA/miRNA Universal Kit according to the protocol “Simultaneous Purification of Genomic DNA and Total RNA, including miRNA, from Cells” (QIAGEN GmbH). Tumor tissue from patients 2 and 4 were excluded due to technical reasons.

### Molecular karyotyping using SNP-array

Depending on the availability, blood samples, tumor tissue, GSCs and CD133^pos.^/CD15^pos.^ cells were subjected to genome-wide copy number variation (CNV) analysis and assessment of copy number neutral loss of heterozygosity (cn-LOH) chromosomal regions using SNP array (Affymetrix CytoScan^®^ 750 Array, ATLAS Biolabs, Berlin, Germany). Genomic DNA was extracted from peripheral blood, tumor tissue, GSCs, and CD133^pos.^/CD15^pos.^ cells according to the protocols “DNA purification from Blood or Body Fluids” or “Isolation of Total DNA from Tissues” from the QIAamp**^®^** DNA Investigator Kit (QIAGEN). DNA quality was checked by agarose gel electrophoresis. SNP array analyses was carried out using the Affymetrix Chromosome Analysis Suite (ChAS 2.0.0.195) *vs* reference data file Affymetrix CytoScan750_Array.na32.3.v., using the copy number and LOH workflows with standard settings. We considered chromosomal regions of CNVs and cn-LOHs ≥ 3 Mb as reliable [14].

### Gene expression analyses

Raw data of all 47,231 gene-expression probes was extracted by Illumina GenomeStudio (Illumina, San Diego, California, United States of America). Data was further processed within R / Bioconductor. All analyses were done in R (R Core Team 2015). False discovery rate and the proportion of null values of all tested hypotheses were calculated based on empirical null modelling and Grenander-density approaches as implemented in the package fdrtool [18]. For Pathway enrichment we used hypergeometric tests. Thereby, we used all 16,010 genes surviving preprocessing in at least on tissue as background. As pathway references we used GO (gene ontology), KEGG (Kyoto Encyclopedia of Genes and Genomes), Reactome, and DOSE (Disease Ontology Semantic and Enrichment) as implemented in packages ReactomePA [19], clusterProfiler [20], and DOSE [21]. Genes for pathway analysis were restricted to FDR 5% level on single-marker analysis and being at least twice times over- and under-expressed. Additional details are available in S1 File.

## Results

### SNP array analysis

Our SNP array analyses detected copy number variations (CNVs) in all donor samples within tumor tissue, GSCs, and CD133^pos.^/CD15^pos.^ cells. It did not detect any CNVs in blood samples. When comparing tumor tissue and cell subpopulations, our analyses mainly revealed a unique genetic profile. For example, a gain of chromosome 7 as well as partial (10q11.1→q26.3 and 10q23.31) and complete loss of chromosome 10 occurred in tumor tissue and cell subpopulations derived from patients 3 and 4.

**Fig 1:**
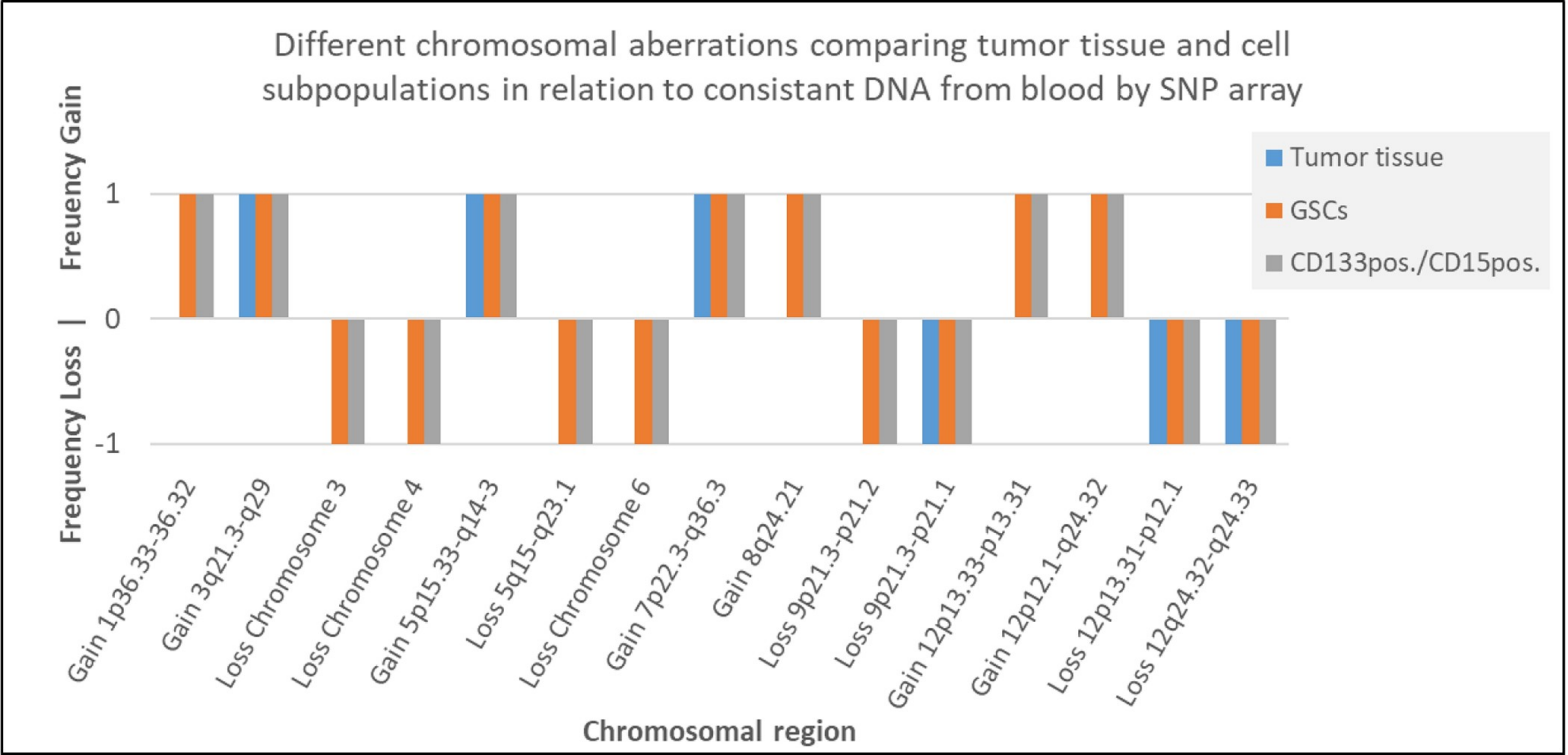
Comparison of chromosomal aberrations between tumor tissue and cell subpopulations.

**Fig 2:**
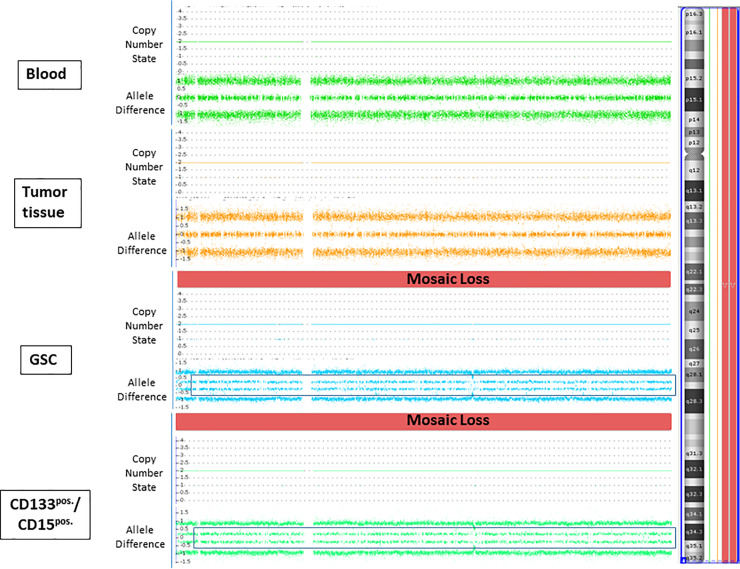
Genetic analyses of peripheral blood, tumor tissue, GSCs and CD133^pos.^/CD15^pos.^ cells. SNP array analyses (CytoScan Array and ChAS software, Affymetrix^®^) of patient 5 revealed a clonal (mosaic) occurrence of copy number variation with loss of chromosome 4 only in cell subpopulations but not in tumor tissue. The heterozygous allele cluster splits into two clusters (shown in black frames) and reflects allele proportions deviating slightly from 1:1.

**Fig 3:**
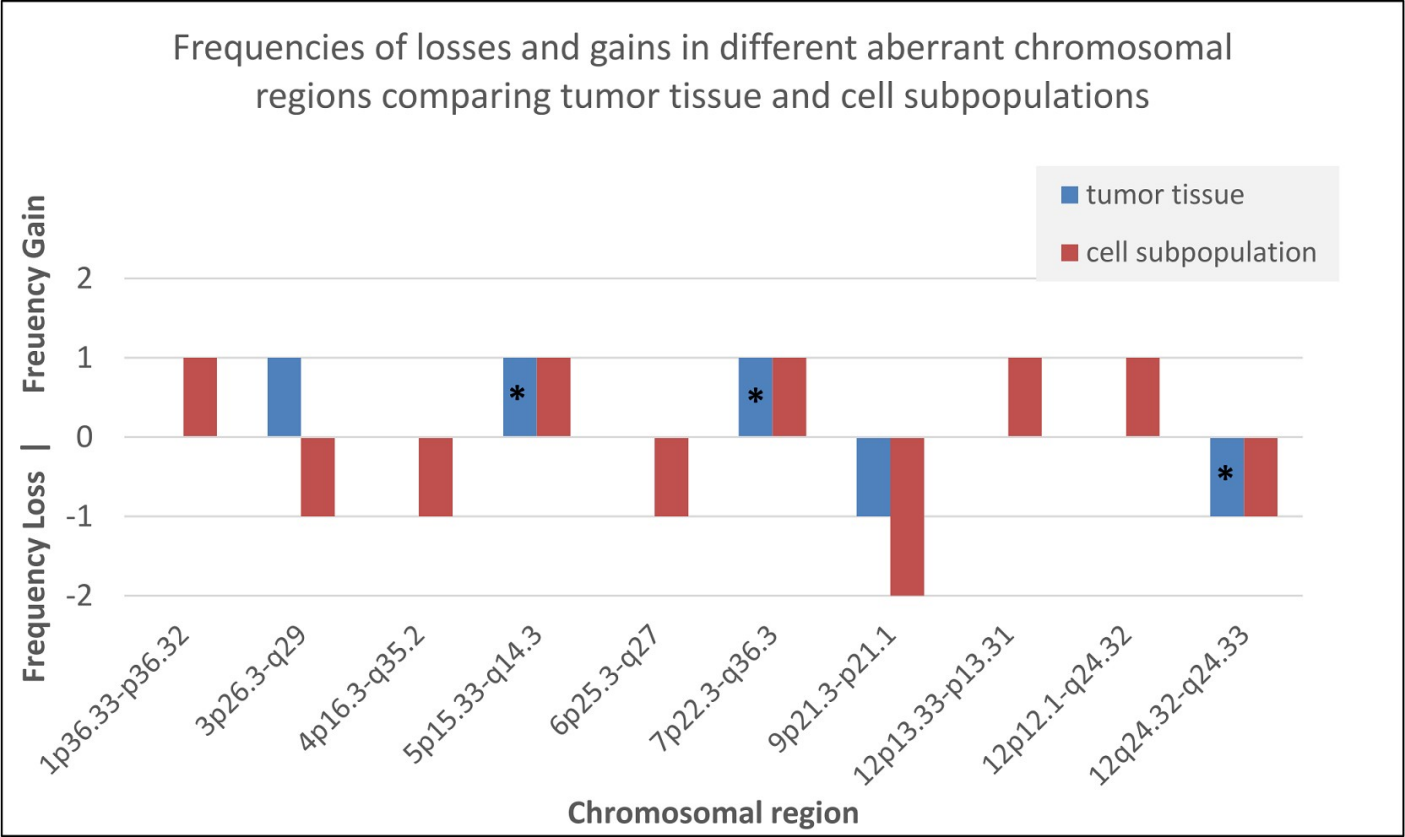
Frequencies of different chromosomal aberrations when comparing tumor tissue and cell subpopulations. Legend: * clonal occurrence of the chromosomal aberration in tumor tissue but not clonal occurrence in cell subpopulations.

However, we also detected 15 distinct genetic differences between tumor tissue and isolated cell subpopulations (Fig 1; S1 Table). Nine of these differences were genetic aberrations that were present in cell subpopulations but not within tumor tissue. These included complete chromosomal loss of chromosomes 3, 4 (example for loss of chromosome 4 is shown in Fig 2) and 6 in one patient. These abnormalities were present only in GSCs and CD133^pos.^/CD15^pos.^ cells. In the remaining genetic anomalies described, clonal occurrence of gain or loss of chromosomal material was detected only in tumor tissue but not in cell subpopulations (S1 Table). Frequencies of losses and gains in different aberrant chromosomal regions comparing tumor tissue and cell subpopulations are shown in Fig 3. According to the Atlas of Genetics and Cytogenetics in Oncology and Haematology, 409 cancer genes were implicated by the 15 genetic aberrations we identified. Of these genes, 49 have been previously described in relation to glioblastoma (S2 Table).

**Fig 4:**
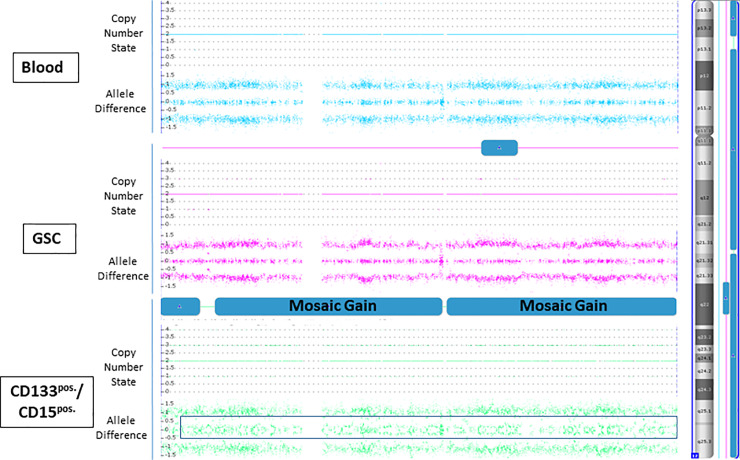
Genetic analyses of peripheral blood, GSCs and CD133^pos.^/CD15^pos.^ cells. In patient 4, SNP array analyses additionally showed a clonal (mosaic) occurrence of copy number variation with gain of chromosome 17 only in CD133^pos.^/CD15^pos.^ cells. The heterozygous allele cluster splits into two clusters (shown in black frame) and reflects allele proportions deviating slightly from 1:1. The blue bar in GSCs give a possible gain signal in the ChAS software in this chromosomal region, but was defined as not gain because other parameters as copy number state and allele difference do not indicate a gain in this chromosomal region.

Furthermore, we identified 16 distinct, not previously described genetic differences between GSCs and CD133^pos.^/CD15^pos.^ cells (S3 Table). For example, the clonal occurrences of loss of chromosome 16 and of gain of chromosome 17 in patient 4 were observed only in CD133^pos.^/CD15^pos.^ cells but not in GSCs (example of gain of chromosome 17 is shown in Fig 4). We detected a gain of chromosomal region 12p13.33-p13.31 (patient 1) as a clonal occurrence only in GSCs, but not clonal in CD133^pos.^/CD15^pos.^cells. Within all these 16 different aberrant chromosomal regions, according to the Atlas of Genetics and Cytogenetics in Oncology and Haematology, 355 cancer genes were identified. 34/355 genes have been previously described in relation to glioblastoma (S4 Table).

Our analyses showed mainly unique cn-LOH regions between tumor tissue and cell subpopulations of patients 1, 3, and 5 at chromosomes 1, 10, 16, and 18. We also detected, in total, seven distinct genetic different cn-LOH regions when comparing tumor tissue and cell subpopulations (S5 Table). According to the Atlas of Genetics and Cytogenetics in Oncology and Haematology, we identified 181 cancer genes within these different cn-LOH regions. Of these, 21 genes have been previously described in relation to glioblastoma (S5 Table). No differences were detected when comparing GSCs and CD133^pos.^/CD15^pos.^ cells.

### Gene expression profiling

We then investigated which genes were different between groups and looked for global differences. We also analyzed individual differences by focusing on the following cell populations: CD133^pos.^/CD15^pos.^ cells vs. tumor tissue, GSCs vs. tumor tissue, CD133^pos.^/CD15^pos.^ cells vs. GSCs.

**Fig 5:**
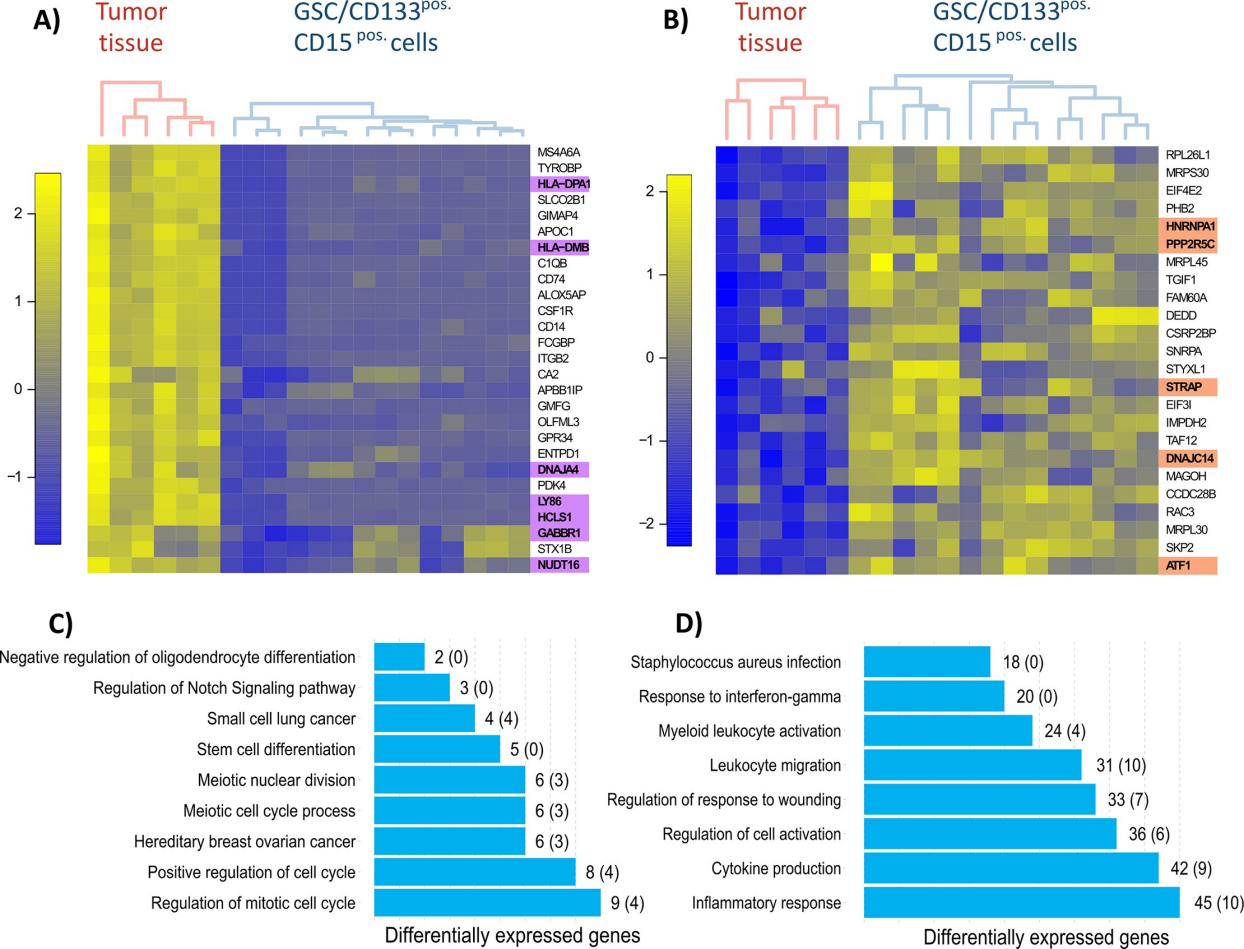
Gene expression profiling of glioblastoma tumor tissue and isolated glioblastoma stem cell populations. The cluster is a hierarchical cluster and a visualization of the distance matrix (pairwise similarity of gene expression levels). In gene expression analyses the two-group test was performed, so that two clusters occurred. Two Illumina chips were applied. A) Top-Genes upregulated (yellow color) in glioblastoma tumor tissue vs. glioblastoma stem cell subpopulation CD133^pos.^/CD15^pos.^ cells. B) Top-Genes upregulated (blue color) in glioblastoma stem cell subpopulation CD133^pos.^/CD15^pos.^ cells vs. glioblastoma tissue. The heat maps show expression levels from all analyzed subpopulations. The cluster is a hierarchical clustering and a visualization of the distance matrix (pairwise similarity of gene expression level). Consequently, similar samples are next to each other. In gene expression analysis we used the two-group comparison, so we stained the samples in two cluster. The algorithm stained the following two most similar groups: tumor tissue vs. CD133^pos.^/CD15^pos.^ cells. C) Detected Pathways by EnrichmentR analyses with number of differentially expressed genes when consider upregulating genes in cell subpopulations vs. tumor tissue. In brackets number of cancer genes are displayed. D) Detected Pathways by EnrichmentR analyses with number of differentially expressed genes when consider upregulating genes in tumor tissue vs. cell subpopulations. In brackets number of cancer genes are displayed.

Our gene expression analyses unveiled uniquely upregulated and downregulated genes in tumor tissue compared to cell subpopulations. From the distribution of *p*-values, we estimate that about 37% of the transcriptome is altered when comparing CD133^pos.^/CD15^pos.^ labeled cells and tumor tissue, and about 30% is altered when comparing GSCs and tumor tissue. When comparing CD133^pos.^/CD15^pos.^ cells with tumor tissue, 418 genes were upregulated in tumor tissue and 44 genes were upregulated in CD133^pos.^/CD15^pos.^ cells with a minimum absolute fold change of two (Fig 5A and 5B).

When comparing our gene expression analyses and genetic analyses, the cancer genes *HLA-DPA1*, *HLA-DMB*, *DNAJA4*, *LY86*, *HCLS1*, *GABBR1*, *NUDT16* showed upregulation in tumor tissue compared to cell subpopulations and are located in chromosomal regions that showed losses in GSC and CD133^pos.^/CD15^pos.^ cells. In contrast, the cancer genes *HNRNPA1*, *PPP2RC5*, *STRAP*, *DNAJC14*, *ATF1* showed upregulation in the cell subpopulations GSC and CD133^pos.^/CD15^pos.^ cells and are located in chromosomal regions that had gains in GSC and CD133^pos.^/CD15^pos.^ cells.

### Pathway analyses

Our pathway analyses showed that upregulated genes in tumor tissue (compared to CD133^pos.^/CD15^pos.^ cells) may influence immune pathways. The most significant, theoretically involved pathways are Staphylococcus aureus infection (*p*-value = 1.49*10^−15^), myeloid leukocyte activation (*p*-value = 6.59*10^−13^), and inflammatory response (*p*-value = 4.41*10^−12^). Pathways of different diseases were also implicated, including demyelinating disease (*p*-value = 1.07*10^−11^) and arteriosclerotic cardiovascular disease (*p*-value = 1.37*10^−10^) (Fig 5D; Table 2). More detailed information are available in S6 Table.

**Table 2:** Implicated cancer genes and their influence on gene pathways upregulated in tumor tissue vs. CD133^pos.^/CD15^pos.^ cells.

Database	Term	p value	Influence on	HGNC cancer genes (
KEGG	Hsa05150	1,49*10^−15^	Staphylococcus aureus infection	-
GO	GO:0002274	6,59*10^−13^	Myeloid leukocyte activation	*CD74*, *SPI1*, *PYCARD*, *TGFBR2*
GO	GO:0006954	4.41*10^−12^	Inflammatory response	*CSF1R*, *CEBPA*, *PYCARD*, *ALOX5*, *SPP1*, *S100A8*, *CASP1*, *S100A9*, *APOE*, *IL1B*
GO	GO:0050865	9,04*10^−11^	Regulation of cell activation	*CD74*, *PYCARD*, *TGFBR2*, *CTGF*, *MERTK*, *IL1B*
GO	GO:1903034	6,47*10^−8^	Regulation of response to wounding	*TGFBR2*, *SPP1*, *S100A8*, *CASP1*, *S100A9*, *PTEN*, *IL1B*
GO	GO:0050900	1,94*10^−10^	Leukocyte migration	*CD74*, *PYCARD*, *SPP1*, *S100A8*, *S100A9*, *CCL3L3*, *CD34*, *MERTK*, *IL1B*, *MMP9*
GO	GO:0034341	3,94*10^−10^	Response to interferon-gamma	-
GO	GO:0001816	6,78*10^−10^	Cytokine production	*CSF1R*, *CD74*, *PYCARD*, *S100A8*, *CASP1*, *S100A9*, *SULF2*, *SULF1*, *IL1B*

The implicated inflammatory processes are likely due to the presence of immune cells in the tissue samples and the lack of immune cells in the cell-specific samples.

In contrast, the upregulated genes in CD133^pos.^/CD15^pos.^ cells (compared to tumor tissue) may mainly influence the regulation of cell cycle process and cancerogenesis. Our analyses showed that the most significant, theoretically involved pathways are positive regulation of cell cycle (*p*-value = 1.59*10^−6^), meiotic cell cycle process (*p*-value = 3.09*10^−6^), and regulation of mitotic cell cycle (p-value = 5.16*10^−6^). Various other cancer pathways are also involved, such as hereditary breast ovarian cancer (*p*-value = 1.30*10^−4^), small cell lung cancer (*p*-value = 3.33*10^−4^), and regulation of Notch signaling (*p*-value = 5,68*10^−4^) (Fig 5C; Table 3). More detailed information are available in S7 Table.

**Table 3:** Implicated cancer genes and their influence on gene pathways upregulated in CD133^pos.^/CD15^pos.^ cells vs. tumor tissue.

Database	Term	P value	Influence on	HGNC cancer genes
GO	GO:0045787	1.59*10^−6^	Positive regulation of cell cycle	*CDKN1B*, *CDK2*, *AURKA*, *UBE2C*
GO	GO:0007126	2.36*10^−6^	Meiotic nuclear division	*CDK2*, *CKS2*, *AURKA*
GO	GO:1903046	3.09*10^−6^	Meiotic cell cycle process	*CDK2*, *CKS2*, *AURKA*
GO	GO:0007346	5.16*10^−6^	Regulation of mitotic cell cycle	*CDKN1B*, *CDK2*, *AURKA*, *UBE2C*
DOSE	DOID:5683	1.30*10^−4^	Hereditary breast ovarian cancer	*CDKN1B*, *CDK2*, *AURKA*
KEGG	Hsa05222	3.33*10^−4^	Small cell lung cancer	*CDKN1B*, *CDK2*, *CKS2*, *CKS1B*
GO	GO:0048863	4.36*10^−4^	Stem cell differentiation	-
GO	GO:0008593	5.68*10^−4^	Regulation of Notch Signaling pathway	-
GO	GO:0048715	5.98*10^−4^	Negative regulation of oligodendrocyte differentiation	-

## Discussion

In the present study, we sought to gain a better understanding of the genetic and transcriptomic landscape of human glioblastoma tissue as well as stem cells isolated from this tissue. We utilized specific stem cell markers to confirm the stemness of our primary cell culture lines and uncovered unique DNA and RNA characteristics of both tumor tissue and GSCs.

We used the markers CD133 and CD15 in combination to label GSCs. Although tumors can grow from CD133^neg.^ cells, the expression of CD133 has been attributed to the aggressiveness of the tumor. Moreover, glioblastoma tumors that recur following radiotherapy or chemotherapy have a higher percentage of CD133^pos.^ cells [22]. However, CD133 expression should be considered with care to enrich any cells because surface CD133 marks stem cells and decreases with differentiation while the expression of *prominin-1* mRNA is not regulated with stemness [23]. This suggests that only the glycosylated surface protein CD133 is cancer stem-like cell-specific. However, it is quite certain that CD133 has a false-negative rate when identifying cancer stem-like cells (CSCs). In addition, CD133^neg.^ cells can be tumor propagating in some tumors and tumor spheres formed by GSCs have also been documented to express CD15 in addition to CD133 [24]. Moreover, both CD133 and CD15 have been shown to define unique subpopulations of stem-like glioblastoma cells [25].

We detected a mainly unique genetic profile in comparison of tumor tissue and cell subpopulation, and these results corroborate prior reports that chromosomal abnormalities are common in glioblastoma and isolated stem-like cells [10,26–29]. Our results showed that it is recommendable to isolate cells with stem cell characters and to define cell subpopulations to get more genetic information. It is possible that the SNP-array analysis did not detect all aberrations in tumor tissue because of the heterogeneity of many cell types in the tumor mass. Chromosomal aberration of the chromosomal region 1p, which we detected only in cell subpopulations but not in tumor tissue, is also known for other tumors like oligodendroglioma [9,14]. In our analyses, the chromosomal region 9p21.3-p21.1, including the gene *CDKN2A*, was different in tumor tissue (1x clonal loss; 1x no aberration) and cell subpopulations (loss). Lathia et al. described in their review *CDKN2A* as one of the tumor suppressors in GBM [9]. The transcription factor NANOG is important for GSC identity according to Lathia et al. [9] and is included in a chromosomal region (12p13.33-p13.31) which was different between tumor tissue (no aberration) and cell subpopulation (gain) in our analyses. Gain of 12p13.33-p13.31 has been also already described in glioblastoma [30], central neurocytoma [14] and neuroblastoma [31,32]. Suva et al. identified a core set of four transcriptional factors in proneural GBM, which were able to reprogram differentiated tumor cells into glioma cancer stem-like cells [33]. It was described that *POU3F2*, *SOX2*, *SALL2*, and *OLIG2* maintain the tumor-forming capability of these cells. In our analyses, these four genes were not different when comparing tumor tissue and cell subpopulations. Kim et al. identified *EZH2* to be important for cancer stem-like cell maintenance because of its regulatory function of both Polycomb-repressive domains and STAT3 signaling [34]. *EZH2* overexpression has been observed in different malignancies, including glioblastoma [35]. In our results chromosome 7, containing *EZH2*, showed a clonal gain in tumor tissue but not clonal gain in cell subpopulations. Suvà et al. demonstrated that disruption of *EZH2* by DZNep, or its specific down-regulation impairs glioblastoma cancer stem cell self-renewal *in vitro* and tumor-initiating capacity *in vivo*. In addition, Suvà et al. showed the direct transcriptional regulation of c-myc by EZH2, suggesting its role as valuable therapeutic target of patients with GBM [36]. Our analyses showed cn-LOH regions at 10q23.3, including *PTEN* and at 17p13.1, including *TP53*. Both these genes (*PTEN* and *TP53*) have been often described as aberrant in glioblastoma.

The cancer genes *MIR200A*, *MIR200B*, and *MIR429* in the different aberrant chromosomal region 1p36.33-p36.32 between tumor tissue (no aberration) and cell subpopulations (gain) have been described as implicated in regulation of tumor cell proliferation in glioma [37]. Homozygous deletion of CDKN2B has been described as a signature genetic event that drives the pathogenesis of GBM. The deletion of this locus is the most common homozygous deletion, present in 75% of GBM samples [38].

Compared to tumor tissue, upregulated genes in CD133^pos.^/CD15^pos.^ cells implicated distinct pathways largely relevant to cell cycle processes and cancerogenesis. These pathways suggest that we probably identified cancer stem cells with a stem cell character. The cancer genes *HNRNPA1*, *PPP2RC5*, *STRAP*, *DNAJC14*, and *ATF1* showed upregulation in GSC and CD133^pos.^/CD15^pos.^ cells and were included in chromosomal regions that showed gains for GSCs and CD133^pos.^/CD15^pos.^cells. We suggest that these genes may be essential for the glioblastoma stem cell population. Supportive of this, the cancer genes *HNRNPA1* and *STRAP* exhibit high expression in diverse cancers. Loh et al. found out that silencing of *HNRNPA1* in MDA-MB-231 cells induced cell death and decreased cell invasion [39]. Otsuka et al. described lower cell proliferation in MDA-MB-231 cells when silencing *HNRNPA1* [40]. Similarly, the protein STRAP promotes tumorigenicity and metastasis in various cancers [41]. It is also known that activated NOTCH signaling promotes the self-renewal capacity of colorectal cancer stem-like cells [42]. Previously, Jin et al. hypothesized that STRAP might participate in the activation of NOTCH signaling and influence the molecular signature of colorectal stem-like cells [41]. These results highlight the potential importance of STRAP for stemness of cells and may also play a role in the proliferation of glioblastoma cells. Future studies are warranted to explore the clinical importance of STRAP.

In summary, we performed basic research on patient matched CD133^pos.^/CD15^pos.^ cells and bulk tumor tissue derived from primary human glioblastoma samples. We identified individual genomic aberrations in human glioblastoma tissue as well as stem-like cells derived from this tissue. We observed individual chromosomal abnormalities between tumor tissue and cellular subpopulations. Moreover, our gene expression profiling analyses uncovered previously undescribed pathways that are upregulated and downregulated in isolated cells compared to tumor tissue. Our analyses of detected upregulated or down regulated genes in gene expression correlated with the detected aberrant chromosomal regions we identified, using SNP array technique. Additional research endeavors should aim to develop more optimal markers for GSCs and to identify robust molecular targets for future therapies. Further analysis of the genes *FABP7* and *STRAP* in GSCs compared to tumor tissue may be especially efficacious. Protein analyses could be helpful to get more information about the mechanism by which FABP7 progresses glioblastoma. A prospective approach could be to test antibodies from overexpressed genes at GBM samples and to compare these results with gene expression patterns to get a prognostic predictive marker. Another idea would be to perform a targeted manipulation (e.g. using siRNA) of glioblastoma cells which show gene overexpression and to track the consequences gene knockdown on the biological behavior of these cells.

## Supporting information

S1 Table Overview of different chromosomal aberrations comparing tumor tissue and cell subpopulations in relation to constant DNA from blood by SNP array. (DOCX)

S2 Table Overview of detected genes involved in glioblastoma of the different aberrant chromosomal regions comparing tumor tissue and cell subpopulations by SNP array. (DOCX)

S3 Table Overview of different chromosomal aberrations comparing GSCs and CD133^pos^/CD15^pos^ cells by SNP array. (DOCX)

S4 Table Overview of detected genes involved in glioblastoma of the different aberrant chromosomal regions comparing GSCs and CD133^pos^/CD15^pos^ cells by SNP array. (DOCX)

S5 Table Overview of differences of cn-LOH regions when comparing tumor tissue and cell subpopulations by SNP array. (DOCX)

S6 Table Involved cancer genes and influence on pathways of genes downregulated in CD133 ^pos^/CD15 ^pos^ cells vs. tumor tissue. (DOCX)

S7 Table Involved cancer genes and influence on pathways of genes upregulated in CD133 ^pos^/CD15 ^pos.^ cells vs. tumor tissue. (DOCX)

S1 File Details for the performed gene expression analyses. (DOCX)
