# Supplementary material for: Molecular analyses of glioblastoma stem-like cells and glioblastoma tissue
Source: PLoS One. 2020 Jul 7;15(7):e0234986. doi: 10.1371/journal.pone.0234986 (PMC7340312; doi:10.1371/journal.pone.0234986)
Supplement: S1 Table — (DOCX) [file pone.0234986.s001.docx]

**S1 Table. Overview of different chromosomal aberrations comparing tumor tissue and cell subpopulations in relation to constant DNA from blood by SNP array**

| **Patient** | **Chromosomal aberration** | **Physical position (Mb)** | **Length (Mbp)** | **Tumor** | **GSCs** | **CD133^pos.^/CD15^pos.^** |
| --- | --- | --- | --- | --- | --- | --- |
| 1 | Gain 1p36.33-p36.32 | 1: 849,466 - 3,190,746 | 2,341 | - | + | + |
| 1 | Gain 3q21.3-q29 | 3: 127,540,576 - 197,851,986 | 70,311 | + | + | + |
|  |  |  |  | Mosaic |  |  |
| 1 | Gain 5p15.33-q14.3 | 5: 113,576 - 90,494,267 | 90,381 | + | + | + |
|  |  |  |  | Mosaic |  |  |
| 1 | Gain 7p22.3-q36.3 | 7: 43,360 - 159,119,707 | 159,076 | + | + | + |
|  |  |  |  | Mosaic |  |  |
| 1 | Loss 9p21.3-p21.1 | 9: 21,293,630 - 30,720,622 | 9,427 | + | + | + |
|  |  |  |  | Mosaic |  |  |
| 1 | Gain 12p13.33-p13.31 | 12: 296,244 - 8,225,225 | 7,929 | - | + | + |
|  |  |  |  |  | Mosaic |  |
| 1 | Loss 12p13.31-p12.1 | 12: 8,567,625 - 24,257,552 | 15,690 | + | + | + |
|  |  |  |  | Mosaic |  |  |
| 1 | Gain 12p12.1-q24.32 | 12: 25,210,123 - 128,011,844 | 102,802 | - | + | + |
|  |  |  |  |  | Mosaic | Mosaic |
| 1 | Loss 12q24.32-q24.33 | 12: 128,486,163 - 133,777,902 | 5,292 | + | + | + |
|  |  |  |  | Mosaic |  |  |
| 3 | Gain 8q24.21 | 8: 127,949,645 - 130,139,748 | 2,190 | - | + | + |
| 5 | Loss Chr. 3 | 3: 61,891 - 197,851,986 | 197,790 | - | + | + |
|  |  |  |  |  | Mosaic | Mosaic |
| 5 | Loss Chr. 4 | 4: 68,345 - 190,957,473 | 190,889 | - | + | + |
|  |  |  |  |  | Mosaic | Mosaic |
| 5 | Loss 5q15-q23.1 | 5: 93,806,931 - 117,285,771 | 23,479 | - | + | + |
| 5 | Loss Chr. 6 | 6: 156,974 - 170,919,482 | 170,763 | - | + | + |
|  |  |  |  |  | Mosaic | Mosaic |
| 5 | Loss 9p21.3-p21.2 | 9: 21,178,270 - 26,571,285 | 5,393 | - | + | + |

Legend:

+: aberration detected

-: no aberration detected
