# Supplementary material for: Molecular analyses of glioblastoma stem-like cells and glioblastoma tissue
Source: PLoS One. 2020 Jul 7;15(7):e0234986. doi: 10.1371/journal.pone.0234986 (PMC7340312; doi:10.1371/journal.pone.0234986)
Supplement: S2 Table — (DOCX) [file pone.0234986.s002.docx]

**S2 Table. Overview of detected genes involved in glioblastoma of the different aberrant chromosomal regions comparing tumor tissue and cell subpopulations by SNP array**

| **Chromosomal**  **region** | **Physical position (Mb)** | **CNV** | | **Described genes in association with glioblastoma** | **Patient** |
| --- | --- | --- | --- | --- | --- |
|  |  | **Tumor tissue** | **Cell**  **subpopulations** |  |  |
| 1p36.33-p36.32 | 1: 849,466 -  3,190,746 | - | gain | *MIR200A, MIR200B, MIR429* | 1 |
| 3p26.3-q29 | 3: 61,891 - 197,851,986 | - | clonal loss | *TGFBR2, ITGA 9, PFKFB4, ROHA, GSK3B* | 5 |
| 3p26.3-q29 | 3: 61,891 - 197,851,986 | clonal gain (1),  - (5) | gain (1),  clonal loss (5) | *PRKCI* | 1,5 |
| 3p26.3-q29 | 3: 61,891 - 197,851,986 | clonal gain (1),  - (5) | gain (1),  clonal loss (5) | *ECT2* | 1,5 |
| 4p16.3-q35.2 | 4: 68,345 - 190,957,473 | - | clonal loss | *EIF4E* | 5 |
| 5p15.33-q14.3 | 5: 113,576 - 90,494,267 | clonal gain | gain | *TRIO, LIFR, PIK3R1* | 1 |
| 6p25.3-q27 | 6: 156,974 - 170,919,482 | - | clonal loss | *AGER, ID4, ROS1, FABP7* | 5 |
| 7p22.3-q36.3 | 7: 43,360 - 159,119,707 | clonal gain | gain | *ACHE, CAV1, EPHA1, ETV1, EZH2, GPNMB, HGF* | 1 |
| 7p22.3-q36.3 | 7: 43,360 - 159,119,707 | clonal gain | gain | *MET, MIR106B, MIR196B, NAMPT, NRCAM, PTN, SEPT7, TWIST1* | 1 |
| 9p21.3-p21.1 | 9: 21,293,630 - 30,720,622 | clonal loss | loss | *TEK* | 1 |
| 9p21.3-p21.1 | 9: 21,293,630 - 30,720,622 | clonal loss (1),  - (5) | loss (1,5) | *CDKN2A, CDKN2B* | 1,5 |
| 12p13.33-p13.31 | 12: 296,244 - 8,225,225 | - | gain | *NANOG* | 1 |
| 12p12.1-q24.32 | 12: 25,210,123 - 128,011,844 | - | clonal gain | *DGKA, PA2G4, GLS2, AGAP2, CDK4, WIF1, MDM2, APAF1, ASCL1, TBX3, HRK* | 1 |
| 12q24.32-q24.33 | 12: 128,486,163 - 133,777,902 | clonal loss | loss | *RAN* | 1 |

Legend:

CNV: copy number variation

-: no aberration detected
