# Supplementary material for: Molecular analyses of glioblastoma stem-like cells and glioblastoma tissue
Source: PLoS One. 2020 Jul 7;15(7):e0234986. doi: 10.1371/journal.pone.0234986 (PMC7340312; doi:10.1371/journal.pone.0234986)
Supplement: S3 Table — (DOCX) [file pone.0234986.s003.docx]

**S3 Table. Overview of different chromosomal aberrations comparing GSCs and CD133^pos.^/CD15^pos.^ cells by SNP array**

| **Patient** | **Chromosomal aberration** | **Physical position (Mb)** | **Length (Mbp)** | **GSCs** | **CD133^pos.^/CD15^pos.^** |
| --- | --- | --- | --- | --- | --- |
| 1 | Gain 12p13.33-p13.31 | 12: 296,244 - 8,225,225 | 7,929 | +  Mosaic | + |
| 2 | Gain 17p13.3-p11.1 | 17: 525 - 25,663,955 | 25,663 | - | +  Mosaic |
| 2 | Gain 17q12-q25.3 | 17: 32,498,819 - 81,041,938 | 48,543 | - | +  Mosaic |
| 4 | Gain 2p25.3-p25.1 | 2: 12,770 - 23,342,002 | 23,329 | - | + |
| 4 | Gain 2p21-p16.3 | 2: 44,327,171 - 48,478,197 | 4,151 | - | + |
| 4 | Loss 2q11.2-q37.3 | 2: 100,169,935 - 242,783,384 | 142,613 | + | +  Mosaic |
| 4 | Gain 3q26.1-q29 | 3: 168,081,696 - 197,851,986 | 29,770 | + | +  Mosaic |
| 4 | Gain 3p26.3-q13.13 | 3:1,289,345 - 106,038,264 | 104,749 | - | +  Mosaic |
| 4 | Loss 8q12.1-q24.3 | 8: 55,421,361 - 146,295,771 | 90,874 | + | +  Mosaic |
| 4 | Gain 12p13.33-p13.31 | 12: 173,786 - 8,865,066 | 8,691 | - | +  Mosaic |
| 4 | Loss 15q11.2-q26.3 | 15: 22,770,421 - 102,330,768 | 79,560 | +  Mosaic | - |
| 4 | Loss Chr. 16 | 16: 988,997 - 90,155,062 | 89,166 | - | +  Mosaic |
| 4 | Gain Chr. 17 | 17: 525 - 81,041,938 | 81,041 | - | +  Mosaic |
| 4 | Loss Chr. 18 | 18: 136,226 - 77,488,440 | 77,352 | - | +  Mosaic |
| 4 | Loss Chr. 19 | 19: 301,638 - 59,040,828 | 58,739 | - | +  Mosaic |
| 4 | Loss 21q11.2-q22.12 | 21: 15,006,457 - 36,843,528 | 21,837 | - | +  Mosaic |

Legend:

Chr.: Chromosome

+: aberration detected

-: no aberration detected
