# Supplementary material for: Molecular analyses of glioblastoma stem-like cells and glioblastoma tissue
Source: PLoS One. 2020 Jul 7;15(7):e0234986. doi: 10.1371/journal.pone.0234986 (PMC7340312; doi:10.1371/journal.pone.0234986)
Supplement: S4 Table — (DOCX) [file pone.0234986.s004.docx]

**S4 Table. Overview of detected genes involved in glioblastoma of the different aberrant chromosomal regions comparing GSCs and CD133pos./CD15pos. cells by SNP array**

| **Chromosomal**  **region** | **Physical position (Mb)** | **CNV** | | **Described genes in association with glioblastoma** | **Patient** |
| --- | --- | --- | --- | --- | --- |
|  |  | **GSC** | **CD133^pos.^/CD15^pos.^** |  |  |
| 2p25.3-p25.1 | 2: 12,770 - 23,342,002 | - | gain | *MYCN* | 4 |
| 2q11.2-q37.3 | 2: 100,169,935 - 242,783,384 | loss | clonal loss | *LRP18, RND3, ITGA6, MIR10B, AAMP* | 4 |
| 3q26.1-q29 | 3: 168,081,696 - 197,851,986 | gain | clonal gain | *PRKCI, ECT2* | 4 |
| 8q12.1-q24.3 | 8: 55,421,361 - 146,295,771 | loss | clonal loss | *BAALC, ENPP2* | 4 |
| 12p13.33-p13.31 | 12: 296,244 - 8,225,225 | mosaic gain (1),  - (4) | gain (1),  clonal gain (4) | *NANOG* | 1,4 |
| 14q11.2-q32.33 | 14: 20,511,672 - 107,285,437 | clonal gain | clonal gain | *NDRG2, BMP4, DIO2, AKT1* | 2 |
| 15q11.2-q26.3 | 15: 22,770,421 - 102,330,768 | clonal loss | - | *MIR211, ADAM10, CTSH* | 4 |
| 16p13.3-q24.3 | 16: 988,997 - 90,155,062 | - | clonal loss | *MAPK3, PYCARD, RBL2, CX3CL1* | 4 |
| 17p13.3-q25.3 | 17: 525 - 81,041,938 | - (2,4) | clonal gain (2,4) | *CRK, TRPV1, TRPV2, ACLY, STAT3, GRN, MIR21, SLC9A3R1, SOCS3* | 2,4 |
| 17p13.3-p11.1 | 17: 525 - 25,663,955 | - | clonal gain | *AURKB, TP53* | 2 |
| 21q11.2-q22.12 | 21: 15,006,457 - 36,843,528 | - | clonal loss | *OLIG2* | 4 |

Legend:

CNV: copy number variation

-: no aberration detected
