# Supplementary material for: Molecular analyses of glioblastoma stem-like cells and glioblastoma tissue
Source: PLoS One. 2020 Jul 7;15(7):e0234986. doi: 10.1371/journal.pone.0234986 (PMC7340312; doi:10.1371/journal.pone.0234986)
Supplement: S5 Table — (DOCX) [file pone.0234986.s005.docx]

**S5 Table. Overview of differences of cn-LOH regions when comparing tumor tissue and cell subpopulations by SNP array**

| **Patient** | **Chromosomal region** | **Physiological position (Mb)** | **Length (Mbp)** | **Blood** | **Tumor**  **tissue** | **GSCs** | **CD133^pos.^/CD15^pos.^** | **Described genes in association with glioblastoma** |
| --- | --- | --- | --- | --- | --- | --- | --- | --- |
| 5 | 6q11.1-q12 | 6: 61,968,745 - 65,384,780 | 3,416 | + | + | Mosaic Loss | Mosaic Loss | - |
| 5 | 9p24.3-p21.3 | 9: 192,128 - 21,073,785 | 20,882 | - | - | + | + | - |
| 5 | 9p21.3-p13.3 | 9: 26,576,085 - 34,895,767 | 8,320 | - | - | + | + | *TEK* |
| 5 | 10p15.3-p11.1 | 10: 95,661 - 38,987,169 | 38,892 | - | Loss | + | + | *ARHGAP21, PFKFB3* |
| 5 | 10q11.21-q26.3 | 10: 42,433,539 - 135,426,536 | 92,993 | - | Loss | + | + | *ADAM12, DOCK1, LGI1, MIR146B, MKI67, MXI1* |
| 5 | 17p13.3-p11.2 | 17: 18,900 - 22,217,883 | 22,199 | - | - | + | + | *AURKB, CRK, PLD2, TP53, TRPV1, TRPV2* |
| 5 | 17q11.1-q25.3 | 17: 25,309,336 - 81,041,938 | 55,733 | - | - | + | + | *ACLY, GRN, MIR21, SLC9A3R1, SOCS3, STAT3* |

Legend:

+: aberration detected

-: aberration not detected
