# Supplementary material for: Molecular analyses of glioblastoma stem-like cells and glioblastoma tissue
Source: PLoS One. 2020 Jul 7;15(7):e0234986. doi: 10.1371/journal.pone.0234986 (PMC7340312; doi:10.1371/journal.pone.0234986)
Supplement: S6 Table — (DOCX) [file pone.0234986.s006.docx]

**S6 Table. Involved cancer genes and influence on pathways of genes downregulated in CD133^pos.^/CD15^pos.^ cells vs. tumor tissue**

| **Contrast** | **Database** | **Term** | **p value** | **FDR**  **(q value)** | **Enrichment** | **Influence on** | **Number of pathway genes differentially expressed** | **Pathway genes differentially expressed** | **HGNC cancer genes** |
| --- | --- | --- | --- | --- | --- | --- | --- | --- | --- |
| Downregulated in CD133^pos.^/CD15^pos.^ cells  vs. tumor tissue | KEGG | Hsa05150 | 1,49E-15 | 2,53E-13 | 11,5 | Staphylococcus aureus infection | 18 | *C1QB, ITGB2, HLA-DMB, HLA-DPA1, HLA-DRB1, HLA-DRA, C1QC, HLA-DRB4, HLA-DOA, HLA-DMA, FCGR1A, FPR3, CFD, FCGR2A, C1S, C3, HLA-DQA1, CFI* | - |
| Downregulated in CD133^pos.^/CD15^pos.^ cells  vs. tumor tissue | GO | GO:0002274 | 6,59E-13 | 1,73E-09 | 6,2 | Myeloid leukocyte activation | 24 | *CD74, TYROBP, DOCK2, AIF1, CX3CR1, FCER1G, SPI1, TLR7, SNCA, LTBR, PYCARD, CD86, VAMP8, TGFBR2, HAMP, CD93, RAC2, PTPRE, LAT2, FGR, LYN, HMOX1, CXCL8, CNR1* | *CD74, SPI1, PYCARD, TGFBR2* |
| Downregulated in CD133^pos.^/CD15^pos.^ cells  vs. tumor tissue | GO | GO:0006954 | 4.41E-12 | 5,80E-09 | 3,2 | Inflammatory response | 45 | *CSF1R, CD14, ITGB2, LY86, AIF1, SERPINA3, HLA-DRB1, CX3CR1, FCER1G, TLR7, CEBPA, PYCARD, ALOX5, CD163, HLA-DRB4, STAB1, VAMP8, SERPINA1, HAMP, SPP1, F3, NOX4, PLA2G4C, A2M, S100A8, CASP1, S100A9, APOE, ADORA1, CCL3L3, CYBA, LYN, LXN, SERPING1, TNFRSF1B, CHI3L1, HMOX1, C3, CXCL8, LY96, IL1B, CNR1, CFI, LIPA, AGT* | *CSF1R, CEBPA, PYCARD, ALOX5, SPP1, S100A8, CASP1, S100A9, APOE, IL1B* |
| Downregulated in CD133^pos.^/CD15^pos.^ cells  vs. tumor tissue | GO | GO:0050865 | 9,04E-11 | 5,94E-08 | 3,4 | Regulation of cell activation | 36 | *CD74, HLA-DMB, HLA-DPA1, AIF1, NCKAP1L, HLA-DRB1, FCER1G, HLA-DRA, PLEK, SNCA, VSIG4, PYCARD, HLA-DRB4, CD86, HLA-DOA, HLA-DMA, VAMP8, TNFSF13B, TGFBR2, HAMP, CTGF, RAC2, PTPRE, HLA-E, CORO1A, C1QTNF1, APOE, FGR, LRRC32, CAPN3, LYN, MERTK, HMOX1, HLA-DQA1, IL1B, CNR1* | *CD74, PYCARD, TGFBR2, CTGF, MERTK, IL1B* |
| Downregulated in CD133^pos.^/CD15^pos.^ cells  vs. tumor tissue | GO | GO:1903034 | 1,32E-10 | 6,47E-08 | 3,6 | Regulation of response to wounding | 33 | *CX3CR1, FCER1G, TLR7, PLEK, HLA-DRB4, OMG, VAMP8, TGFBR2, HAMP, SPP1, F3, A2M, S100A8, C1QTNF1, CASP1, S100A9, APOE, ADORA1, CAPN3, CCL3L3, ENPP4, LYN, PTEN, CD34, SERPING1, TNFRSF1B, C3, IL1B, FAM46A, CNR1, CFI, HOPX, AGT* | *TGFBR2, SPP1, S100A8, CASP1, S100A9, PTEN, IL1B* |
| Downregulated in CD133^pos.^/CD15^pos.^ cells  vs. tumor tissue | GO | GO:0050900 | 1,94E-10 | 7,28E-08 | 3,8 | Leukocyte migration | 31 | *CD74, ITGB2, AIF1, NCKAP1L, CX3CR1, FCER1G, CSF3R, MAG, DOCK8, PYCARD, PTPRO, SPP1, RAC2, SLC7A7, CORO1A, S100A8, ESAM, COL1A1, PECAM1, S100A9, ADORA1, ATP1B1, CCL3L3, LYN, CD34, MERTK, HMOX1, CXCL8, IL1B, MMP9, CXCL16* | *CD74, PYCARD, SPP1, S100A8, S100A9, CCL3L3, CD34, MERTK, IL1B, MMP9* |
| Downregulated in CD133^pos.^/CD15^pos.^ cells  vs. tumor tissue | GO | GO:0034341 | 3,94E-10 | 1,29E-07 | 5,6 | Response to interferon-gamma | 20 | *HLA-DPA1, AIF1, HLA-DRB1, IRF8, HLA-DRA, SNCA, HLA-DRB4, CD86, IFI30, FCGR1A, HLA-E, IRF9, CCL3L3, PARP9, BST2, HLA-DQA1, GBP2, HLA-B, IFITM2, CXCL16* | - |
| Downregulated in CD133^pos.^/CD15^pos.^ cells  vs. tumor tissue | GO | GO:0001816 | 6,78E-10 | 1,87E-07 | 2,9 | Cytokine production | 42 | *CSF1R, CD74, CD14, HLA-DPA1, NCKAP1L, HLA-DRB1, IRF8, FCER1G, TLR7, PLCG2, VSIG4, SRGN, CARD9, PYCARD, HLA-DRB4, CD86, HLA-E, S100A8, CASP1, S100A9, IRF9, FGR, LRRC32, TGFB3, CYBA, LYN, SULF2, CD34, SULF1, RGCC, CHI3L1, HMOX1, BST2, C3, ZFP36, LY96, IL1B, ABCA1, HLA-B, HSPA1A, LIPA, AGT* | *CSF1R, CD74, PYCARD, S100A8, CASP1, S100A9, SULF2, SULF1, IL1B* |

**Abbrevations:** KEGG, Kyoto Encyclopedia of Genes and Genomes; GO, Gene Ontology; FDR, false discovery rate; HGNC, HUGO Gene Nomenclature Committee
