# Supplementary material for: Molecular analyses of glioblastoma stem-like cells and glioblastoma tissue
Source: PLoS One. 2020 Jul 7;15(7):e0234986. doi: 10.1371/journal.pone.0234986 (PMC7340312; doi:10.1371/journal.pone.0234986)
Supplement: S7 Table — (DOCX) [file pone.0234986.s007.docx]

**S7 Table. Involved cancer genes and influence on pathways of genes upregulated in CD133^pos.^/CD15^pos.^ cells vs. tumor tissue**

| **Contrast** | **Database** | **Term** | **p value** | **FDR**  **(q value)** | **Enrichment** | **Influence on** | **Number of pathway genes differentially expressed** | **Pathway genes differentially expressed** | **HGNC cancer genes** |
| --- | --- | --- | --- | --- | --- | --- | --- | --- | --- |
| Upregulated in CD133^pos.^/CD15^pos.^ cells vs. tumor tissue | GO | GO:0045787 | 1.59E-06 | 7,27E-04 | 9,4 | Positive regulation of cell cycle | 8 | *CDKN1B, CDK2, CDC25B, AURKA, UBE2C, HES1, LEF1, NUSAP1* | *CDKN1B, CDK2, AURKA, UBE2C* |
| Upregulated in CD133^pos.^/CD15^pos.^ cells vs. tumor tissue | GO | GO:0007126 | 2.36E-06 | 7,27E-04 | 15,2 | Meiotic nuclear division | 6 | *CDK2, CCNB1IP1, CKS2, CDC25B, AURKA, PTTG3P* | *CDK2, CKS2, AURKA* |
| Upregulated in CD133^pos.^/CD15^pos.^ cells vs. tumor tissue | GO | GO:1903046 | 3.09E-06 | 7,27E-04 | 14,6 | Meiotic cell cycle process | 6 | *CDK2, CCNB1IP1, CKS2, CDC25B, AURKA, PTTG3P* | *CDK2, CKS2, AURKA* |
| Upregulated in CD133^pos.^/CD15^pos.^ cells vs. tumor tissue | GO | GO:0007346 | 5.16E-06 | 8,97E-04 | 6,7 | Regulation of mitotic cell cycle | 9 | *CDKN1B, CDK2, CDC25B, AURKA, PTTG3P, UBE2C, HES1, GAS1, NUSAP1* | *CDKN1B, CDK2, AURKA, UBE2C* |
| Upregulated in CD133^pos.^/CD15^pos.^ cells vs. tumor tissue | DOSE | DOID:5683 | 1.30E-04 | 2,64E-02 | 7,3 | Hereditary breast ovarian cancer | 6 | *HNRNPA1, FASN, CDKN1B, CDK2, AURKA, UBE2T* | *CDKN1B, CDK2, AURKA* |
| Upregulated in CD133^pos.^/CD15^pos.^ cells vs. tumor tissue | KEGG | Hsa05222 | 3.33E-04 | 2,21E-02 | 11,7 | Small cell lung cancer | 4 | *CDKN1B, CDK2, CKS2, CKS1B* | *CDKN1B, CDK2, CKS2, CKS1B* |
| Upregulated in CD133^pos.^/CD15^pos.^ cells vs. tumor tissue | GO | GO:0048863 | 4.36E-04 | 1,51E-02 | 7,7 | Stem cell differentiation | 5 | *CORO1C, TEAD2, HEY2, HES1, LEF1* | - |
| Upregulated in CD133^pos.^/CD15^pos.^ cells vs. tumor tissue | GO | GO:0008593 | 5.68E-04 | 1,59E-02 | 18,4 | Regulation of Notch Signaling pathway | 3 | *DLX1, HEY2, HES1* | - |
| Upregulated in CD133^pos.^/CD15^pos.^ cells vs. tumor tissue | GO | GO:0048715 | 5.98E-04 | 1,59E-02 | 54,2 | Negative regulation of oligodendrocyte differentiation | 2 | *DLX1, HES1* | - |

**Abbrevations:** KEGG, Kyoto Encyclopedia of Genes and Genomes; GO, Gene Ontology; FDR, false discovery rate; HGNC, HUGO Gene Nomenclature Committee
