# Supplementary material for: Molecular analyses of glioblastoma stem-like cells and glioblastoma tissue
Source: PLoS One. 2020 Jul 7;15(7):e0234986. doi: 10.1371/journal.pone.0234986 (PMC7340312; doi:10.1371/journal.pone.0234986)
Supplement: S1 File — (DOCX) [file pone.0234986.s008.docx]

**Gene expression analysis**

Raw data of all 47,231 gene-expression probes was extracted by Illumina GenomeStudio (Illumina, San Diego, California, United States of America) without additional background correction. Data was further processed within R / Bioconductor. Expression values were log2-transformed and quantile-normalised [1]. Batch effects of expression BeadChips were corrected using an empirical Bayes method [2]. Within pre-processing, gene-expression probes that were not expressed in at least 5% in a subgroup of the samples were excluded as well as probes still found to be significantly associated with batch effects after Bonferroni-correction. Thereby, considered subgroups were tumor tissue, GSC-cells, sorted CD133^+^/CD15^+^ GSC cells, sorted CD133^-^/CD15^-^ GSC cells. Furthermore, gene-expression probes with poor mapping on the human transcriptome [3] were also excluded. These filters resulted in 25,442 valid gene-expression probes corresponding to 16,010 unique genes found in at least one subgroup.

Three criteria were used to remove samples of low quality: First, the number of detected gene-expression probes of a sample was required to be within ± 4 interquartile ranges (IQR) from the median. Second, the Mahalanobis distance of several quality characteristics of each sample signal of Ambion(TM) ERCC Spike-In control probes, signal of biotin-control-probes, signal of low-concentration control probes, signal of medium-concentration control probes, signal of mismatch control probes, signal of negative control probes and signal of perfect-match control probes [4] had to be within median + 7 x IQR. Third, Euclidean distances of expression values as described [5] had to be within 4 x IQR from the median. Overall, of the assayed 24 samples, 4 samples were excluded for quality reasons.

We used the R add-on package limma [6] to identify differentially expressed genes between all subgroups. Thereby, patient ID was used as blocking factor in order to account for the paired nature of the data. False discovery rate and the proportion of null values of all tested hypotheses were calculated based on empirical null modeling and Grenander-density approaches as implemented in the package fdrtool [7]. For Pathway enrichment we used hypergeometric tests. Thereby, we used all 16,010 genes surviving preprocessing in at least on tissue as background. As pathway references we used GO (gene ontology), KEGG (Kyoto Encyclopedia of Genes and Genomes), Reactome, and DOSE (Disease Ontology Semantic and Enrichment) as implemented in packages ReactomePA [8], clusterProfiler [9], and DOSE [10]. Genes for pathway analysis were restricted to FDR 5% level on single-marker analysis and being at least twice times over- and under-expressed.

**Supplementary References**

1. Schmid R, Baum P, Ittrich C, Fundel-Clemens K, Huber W, Brors B, et al. Comparison of normalization methods for Illumina BeadChip HumanHT-12 v3. BMC Genomics. 2010;11:349. Epub 2010/06/08. doi: 10.1186/1471-2164-11-349. PubMed PMID: 20525181; PubMed Central PMCID: PMCPMC3091625.

2. Johnson WE, Li C, Rabinovic A. Adjusting batch effects in microarray expression data using empirical Bayes methods. Biostatistics. 2007;8(1):118-27. Epub 2006/04/25. doi: 10.1093/biostatistics/kxj037. PubMed PMID: 16632515.

3. Barbosa-Morais NL, Dunning MJ, Samarajiwa SA, Darot JF, Ritchie ME, Lynch AG, et al. A re-annotation pipeline for Illumina BeadArrays: improving the interpretation of gene expression data. Nucleic Acids Res. 2010;38(3):e17. Epub 2009/11/20. doi: 10.1093/nar/gkp942. PubMed PMID: 19923232; PubMed Central PMCID: PMCPMC2817484.

4. Cohen Freue GV, Hollander Z, Shen E, Zamar RH, Balshaw R, Scherer A, et al. MDQC: a new quality assessment method for microarrays based on quality control reports. Bioinformatics. 2007;23(23):3162-9. Epub 2007/10/16. doi: 10.1093/bioinformatics/btm487. PubMed PMID: 17933854.

5. Du R, Lu KV, Petritsch C, Liu P, Ganss R, Passegue E, et al. HIF1alpha induces the recruitment of bone marrow-derived vascular modulatory cells to regulate tumor angiogenesis and invasion. Cancer Cell. 2008;13(3):206-20. Epub 2008/03/11. doi: 10.1016/j.ccr.2008.01.034. PubMed PMID: 18328425; PubMed Central PMCID: PMCPMC2643426.

6. Ritchie ME, Phipson B, Wu D, Hu Y, Law CW, Shi W, et al. limma powers differential expression analyses for RNA-sequencing and microarray studies. Nucleic Acids Res. 2015;43(7):e47. Epub 2015/01/22. doi: 10.1093/nar/gkv007. PubMed PMID: 25605792; PubMed Central PMCID: PMCPMC4402510.

7. Klaus B, Strimmer K. Signal identification for rare and weak features: higher criticism or false discovery rates? Biostatistics. 2013;14(1):129-43. Epub 2012/09/11. doi: 10.1093/biostatistics/kxs030. PubMed PMID: 22962499.

8. Yu G, He QY. ReactomePA: an R/Bioconductor package for reactome pathway analysis and visualization. Mol Biosyst. 2016;12(2):477-9. Epub 2015/12/15. doi: 10.1039/c5mb00663e. PubMed PMID: 26661513.

9. Yu G, Wang LG, Han Y, He QY. clusterProfiler: an R package for comparing biological themes among gene clusters. OMICS. 2012;16(5):284-7. Epub 2012/03/30. doi: 10.1089/omi.2011.0118. PubMed PMID: 22455463; PubMed Central PMCID: PMCPMC3339379.

10. Yu G, Wang LG, Yan GR, He QY. DOSE: an R/Bioconductor package for disease ontology semantic and enrichment analysis. Bioinformatics. 2015;31(4):608-9. Epub 2015/02/14. doi: 10.1093/bioinformatics/btu684. PubMed PMID: 25677125.
